# The ENDS of assumptions: an online tool for the epistemic non-parametric drug–response scoring

**DOI:** 10.1093/bioinformatics/btac217

**Published:** 2022-04-07

**Authors:** Ali Amiryousefi, Bernardo Williams, Mohieddin Jafari, Jing Tang

**Affiliations:** Research Program in Systems Oncology, Faculty of Medicine, University of Helsinki, Helsinki, Finland; Research Program in Systems Oncology, Faculty of Medicine, University of Helsinki, Helsinki, Finland; Research Program in Systems Oncology, Faculty of Medicine, University of Helsinki, Helsinki, Finland; Research Program in Systems Oncology, Faculty of Medicine, University of Helsinki, Helsinki, Finland

## Abstract

**Motivation:**

The drug sensitivity analysis is often elucidated from drug dose–response curves. These curves capture the degree of cell viability (or inhibition) over a range of induced drugs, often with parametric assumptions that are rarely validated.

**Results:**

We present a class of non-parametric models for the curve fitting and scoring of drug dose–responses. To allow a more objective representation of the drug sensitivity, these epistemic models devoid of any parametric assumptions attached to the linear fit, allow the parallel indexing such as half-maximal inhibitory concentration and area under curve. Specifically, three non-parametric models including spline (npS), monotonic and Bayesian and the parametric logistic are implemented. Other indices including maximum effective dose and drug–response span gradient pertinent to the npS are also provided to facilitate the interpretation of the fit. The collection of these models is implemented in an online app, standing as useful resource for drug dose–response curve fitting and analysis.

**Availability and implementation:**

The ENDS is freely available online at https://irscope.shinyapps.io/ENDS/ and source codes can be obtained from https://github.com/AmiryousefiLab/ENDS.

**Supplementary information:**

Supplementary data are available at *Bioinformatics* online.

## 1 Introduction

One of the main goals in toxicology studies is elucidation of the efficacy of a drug with the help of the drug dose–response curves. These curves are the outcome of a statistical fit to percentage viability (or inhibition) as response variable, over the dose concentrations of a drug as the explanatory variable. Most statistical methodology for these studies have been based on the parametric models such as log-logistic, Gaussian, Weibull or Gompertz functions (Holland-Letz and Kopp-Schneider, 2015). The most frequently used method has been the four parameter logistic model by weighted least squares (pL) (Vølund, 1978). This model, albeit powerful, relies on number of assumptions such as normality of residuals which is often unmet in the under sampled studies. Also, the continuity of the explanatory variable is another assumption that is clearly challenged with the pre-defined dose concentrations on a drug dose range which is opposing the goodness of the fit. (Montgomery *et al.*, 2021). These violations of assumptions, not only limit the application of the parametric models, but also adversely affect the derived downstream indices such as half-maximal inhibitory concentration (IC_50_) and area under curve (AUC), as the important measures in determination of the drug efficacy, e.g. in drug synergy testing (Tang *et al.*, 2015). This has implied the application of unimodality and homoscedasticity as alternative measures for drug sensitivity evaluation (Jafari *et al.*, 2021). Despite the common use of the pL in the drug–response scoring, facilitated with number of developed R packages [e.g. drc (Ritz *et al.*, 2015)] the online web application can further eliminate the challenges for the less advanced users. To provide a more objective characterization of the drug sensitivity and ease of use, we propose an online collection of non-parametric drug–response scoring (ENDS) models and indices. This class of models bereft of assumptions regarding the model fitting and its parameters, is appealing for its simplicity and intuitiveness, allowing a more objective characterization of the experiment (epistemic). As such, these models pivot on the results of the experiment and expand more room for enhanced intrinsic data exploration, rather than compromising the accuracy in case of deviation from the underlying assumptions of the parametric models.

## 2 Materials and methods

The ENDS web app is coded in R and is freely accessible at https://irscope.shinyapps.io/ENDS/. Following the Github link provided above, the source code is available for more versatile use by advanced users. The web app also provides the detailed theoretical accounts of the implemented models and related indices (Supplementary Fig. S1). A single-cell level colorectal cancer drug dose–response dataset is used for illustrating the functionality of the tool (Roerink *et al.*, 2018).

### 2.1 Models, data input and usage

**Fig. 1.**
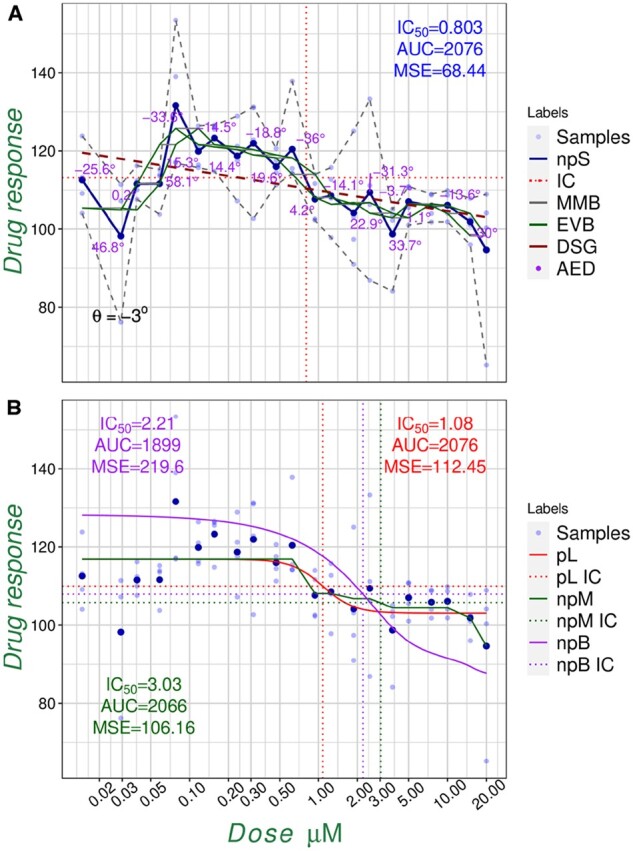
Examples of the ENDS model fitting. The overall outputs of the ENDS models overlaid with the respective IC_50_, AUC and MSE for the 5-FU drug of the first patient sample of the colorectal cancer study (Roerink *et al.*, 2018). Due to different mathematical formulas for each fit, each model is providing a unique IC_50_, AUC and MSE values. (**A**) The npS model on the mean of the samples, absolute efficiency degrees (AEDs), min–max and empirical viability bands (MMB, EVB), next to the DSG ($\theta =-3^{{}^{\circ}}$) and maximum effective dose (MED = 0.85 μM with $-33.6^{{}^{\circ}}$ gradient). (**B**) The npM, npB and pL fits for the same data

The ENDS encompasses four models: (i) non-parametric spline (npS), which connects the mean or median of each dose–response. This simple model is accompanied with novel indices including drug span gradient and min–max viability band (MMB) that facilitate more characterization from the fit (Supplementary Sections S1.1.1 and S1.2.3, Fig. 1A). (ii) Non-parametric monotonic (npM), which is an isotonic regression fit of the npS with non-increasing condition [or non-decreasing in case of percentage inhibition (Supplementary Section S1.1.2; Fig. 1B). (iii) Non-parametric Bayesian (npB), which is a heuristic model fit by a normal cumulative distribution function with a choice of a prior distribution (Supplementary Section S1.1.3; Fig. 1B). (iv) Parametric logistic (pL), which is a conventional four parameter sigmoid fit (Supplementary Section S1.1.4; Fig. 1B). The input of the ENDS is a single .*csv* file including doses as rows (and drug names in the case of multiple drugs) and samples as columns (Supplementary Section S1.2.1). The ENDS by default will generate the npS. Users can add npM, npB and pL model fits and choose the display of more indices e.g. with outliers exclusion option off or a spectrum choice of IC values (Supplementary Section S1.2.3).

## 3 Results

Emphasizing on the non-parametric paradigm, the ENDS provides an online platform for drug dose–response scoring and model fitting. Three fundamentally important yet intrinsically different non-parametric models are introduced that along with other optional scores such as choice of median for each sample, provide a useful tool for drug dose–response analysis. The ENDS also provides the resultant high resolution downloadable plots for single as well as multiple drugs in different formats (Supplementary Sections S1.2.2 and S1.2.3; Fig. 1).

## 4 Discussion and conclusion

The ENDS presents mathematically justified models and have a rigorous theory that supports their use. Despite their non-parametric nature, each model is exhibiting various degrees of simplicity and is intrinsically different. At one end, the npS is intuitive with no internal assumption, while the more heuristic npB is rather computationally costly and may be biased by the choice of prior for small datasets (Supplementary Section S1.1.1). The ENDS also provides the pL fitting and scores. This allows to compare the performance of the models *a posteriori*. For example, our survey over all the available data of the colorectal study (Roerink *et al.*, 2018) revealed the least mean IC_50_ and mean square error (MSE) of the npS fits. While the former indicates the statistically significant over-estimation of the IC_50_ by pL and npB (Supplementary Table S2), the latter is indicating the significantly better fit to the data (Supplementary Table S3). Observing no significant difference between the means of the AUC for different models (Supplementary Table S4) next to the least AUC variance for npB, prioritizes this model over the rest; however, this is challenged with the lower mean and variance MSE of the other models (Supplementary Fig. S2). Despite difference in performance, it is the experimental settings and the level of plausibility of each model that shall guide the users with their preferences. For non-parametric modeling, we believe that the ENDS can stand as a valuable tool to complement the existing applications.

## Funding

We thank the European Research Council (ERC) starting grant DrugComb (Informatics approaches for the rational selection of personalized cancer drug combinations, No. 716063) and Academy of Finland [332454] for the financial support.

*Conflict of* *Interest*: none declared.

### Author Contributions

AA conceived the study and developed the models, AA and BW adopted and implemented the methods, JT provided the funding, AA, BW, MJ, and JT wrote the paper.

## Supplementary Material

btac217_Supplementary_Data

## References

[btac217-B1] Holland-Letz T. , Kopp-Schneider A. (2015) Optimal experimental designs for dose–response studies with continuous endpoints. Arch. Toxicol., 89, 2059–2068. 25155192 10.1007/s00204-014-1335-2 PMC4655015

[btac217-B2] Jafari M. et al (2021) Bipartite network models to design combination therapies in acute myeloid leukaemia. *Nat. Commun*. https://doi.org/10.1038/s41467-022-29793-5. 10.1038/s41467-022-29793-5 PMC9018865 35440130

[btac217-B3] Montgomery D.C. et al (2021) Introduction to Linear Regression Analysis. John Wiley & Sons, USA.

[btac217-B4] Ritz C. et al (2015) Dose-response analysis using R. PLoS One, 10, e0146021. 26717316 10.1371/journal.pone.0146021 PMC4696819

[btac217-B5] Roerink S.F. et al (2018) Intra-tumour diversification in colorectal cancer at the single-cell level. Nature, 556, 457–462. 29643510 10.1038/s41586-018-0024-3

[btac217-B6] Tang J. et al (2015) What is synergy? The Saariselkä agreement revisited. Front. Pharmacol., 6, 181. 26388771 10.3389/fphar.2015.00181 PMC4555011

[btac217-B7] Vølund A. (1978) Application of the four-parameter logistic model to bioassay: comparison with slope ratio and parallel line models. Biometrics, 34, 357–365. 719119

