# Supplementary material for: The ENDS of assumptions: an online tool for the epistemic non-parametric drug–response scoring
Source: Bioinformatics. 2022 Apr 7;38(11):3132–3. doi: 10.1093/bioinformatics/btac217 (PMC9154283; doi:10.1093/bioinformatics/btac217)
Supplement: btac217_Supplementary_Data [file btac217_supplementary_data.pdf]

1 Supplementary Material

In this section we will go through the technical details of the ENDS, the functionality of the web application available at https://irscope.shinyapps.io/ENDS/, a detailed technical explanation of the models and a comparative analysis which was obtained by running the models through the collection of drug-response data found in (Roerink **et al.**, 2018).

1.1 Models

This section provides the mathematical account of the four models presented in the paper.

1.1.1 Nonparametric Spline (*npS*)

The model is the collection of linear functions that connect the average responses at each dose. Given a sample of doses $x_1, .., x_n$ where $x_i \leq x_{i+1}$ and for each i-th dose we have m responses $y_{i1}, .., y_{im}$, then for each dose we can obtain the dose mean $y_i = \frac{1}{m}\sum_{j=1}^{m} y_{ij}$ or alternatively we can calculate the dose medians. The simple spline that connects each of the means with a linear function is given by the piece-wise linear function

$$f(x) = \left(\frac{y_{i+1} - y_i}{x_{i+1} - x_i}\right) x + y_i \quad \text{for } x_i \leq x \leq x_{i+1}.$$

The function is defined on the interval $x_1 \leq x \leq x_n$. Note that this function is not monotonic necessarily, so if we define the IC_{50} as the value in the x-axis such that $f(IC_{50}) = (\max y + \min y)/2$, then it might not be unique as it crosses the above function on multiple points, if the function is constant on an interval I such that $f(I) = (\max y + \min y)/2$, then $\text{IC}_{50} = \inf I$ is the infimum of the values on the interval.

Thus, we calculate all the possible x-axis values that map to the halfway point of the responses and select as the effective dose 50% the one closest in absolute value to the one found by the monotonic fit. For any value $p \in (0,1)$ the IC_p will be the chosen out of the candidates $f(IC_p) = \min y + p(\max y - \min y)$, such that it is closest in distance to the uniquely value obtained by the monotone fit. Note that, if the function is constant we again choose as the IC_p the infinum of the interval.

In order to obtain the angles associated to the slope at each dose we have the ith angle as $\theta_i = arctan((y_{i+1} - y_i)/(x_{i+1} - x_i))$. Note that if the npS is calculated on the means at each dose then the squared error $\frac{1}{nm}\sum_{ij}(y_{ij} - f(x_i))^2$ will be minimized since the mean minimizes the L2 norm, alternatively if we calculate npS using the medians then the L1 norm would be minimized.

1.1.2 Nonparametric Monotone (*npM*)

This nonparametric model is fit over the mean responses at each dose or the medians and it is analogous to npS, with the difference of forcing a non-increasing constrain on the connected linear functions. If the spline between two doses does not have a non-positive slope, then the average of the previous doses is recursively calculated until the next spline has a non-positive slope, which connects previously calculated average with the next data point.

$$f(x) = \begin{cases} \left(\frac{y_{i+1} - y_i}{x_{i+1} - x_i}\right) x + y_i & \text{if } \left(\frac{y_{i+1} - y_i}{x_{i+1} - x_i}\right) \leq 0, \\ \left(\frac{y_{i+1} - \overline{y}}{x_{i+1} - x_i}\right) x + \overline{y} & \text{where } \overline{y} = \frac{1}{k+1}(y_i + .. + y_{i-k}) \end{cases}$$

where k is the smallest integer such that the slope is non-positive, this is $\left(\frac{y_{i+1} - \overline{y}}{x_{i+1} - x_i}\right) \leq 0$. It has been shown that this fit is the one that minimizes the squared errors $\|y - f(x)\|^2$ with the constrain that $f(x_i) \geq f(x_{i+1})$. This fit will produce a non increasing fit, even in cases where the pL model or the npB produce an increasing fit.

For the calculation of the p-th percent max inhibitory concentration $p \in (0,1)$ we find the unique value in the x-axis such that

$$f(IC_p) = \min_{x_1 \leq x \leq x_n} f(x) + p\left(\max_{x_1 \leq x \leq x_n} f(x) - \min_{x_1 \leq x \leq x_n} f(x)\right).$$

1.1.3 Nonparametric Bayesian (*npB*)

This nonparametric model connects a mixture of normal CDFs with Bayesian modeling to solve for the posterior parameters. The parameters knots K and variance λ are given *a priori*. K regulates where the distribution functions will be centered and λ the variance of the distributions, we choose K to be the doses available and $lambda$ is found by grid search, chosen as the one with least square error. Namely, given the points (x_i, y_i) for $i = 1..n$, with knots $K = \{k_i\}_{i=1}^J$ and parameter $\lambda > 0$ we will model the relation between x and y with the Bayesian hierarchical model;

$$C \sim \text{Beta}(1,1)$$
$$a \sim \text{Dir}(\alpha_1, .., \alpha_J)$$
$$\sigma^2 \sim \chi^2(2)$$
$$Y|C, a, \sigma^2, K, \lambda \sim N(f(x), \sigma^2),$$

where

$$f(x) = C + (1 - C)\sum_{j=1}^{J} a_j(1 - F(x|k_j, \lambda)).$$

The weights $a = [a_1, .., a_n]$ are non-negative and sum up to one, and F is the cumulative distribution function of a Gaussian random variable with mean k_j and variance λ. We can use MCMC to sample from the posterior distribution

$$p(C, a, \sigma^2 \mid y, x, K, \lambda)$$

An in-house implementation of the Metropolis-Hastings (MH) sampling algorithm is used for posterior inference, available from the AmiryousefiLab/ENDS Github. Knots are chosen at the unique doses and the parameter λ is chosen as the value with minimum squared error over a grid of values including the mean variance estimate of the samples at each knot. We also chose a uniform Dirichlet distribution as prior for a, this is $a \sim Dir(1, .., 1)$, slightly different model choice from (Roerink **et al.**, 2018) where a stick breaking process is used to generate the weights of the Dirichlet distribution, note that a stick breaking process would force the weights to be decreasing in magnitude with probability one, we think this assumption is too restrictive, so our approach assumes a uniform Dirichlet prior which allows the weights to be any magnitude as long as they are non negative and sum up to one.

Once the samples of the posterior distribution are obtained from the chains of MH algorithm, we drop out the first half of the observations by default. Once we have the estimate of the parameters $\hat{C}$, $\hat{a}$, $\hat{\sigma}^2$ as the mean values, the posterior curve is obtained as

$$\hat{f}(x) = \hat{C} + (1 - \hat{C})\sum_{j=1}^{J} \hat{a}_j(1 - F(x|k_j, \lambda)).$$

The IC_{50} value is calculated from this function as

$$f(IC_{50}) = \frac{1}{2}\left(\max_{x_1 \leq x \leq x_n} f(x) + \min_{x_1 \leq x \leq x_n} f(x)\right),$$

and for any $p \in (0,1)$ we have IC_p obtained as the value such that

$$f(IC_p) = \min_{x_1 \leq x \leq x_n} f(x) + p\left(\max_{x_1 \leq x \leq x_n} f(x) - \min_{x_1 \leq x \leq x_n} f(x)\right).$$

Note that the Bayesian approach would allow us to obtain for each sample of parameters an IC and then have a distribution for it, from which we could take the mean, but to keep the congruency with other models, we opt to obtain the IC_{50} as the cross point of the fit that would be marked over the curve in the plot.

The MH algorithm uses as priors normal distributions with parameters manually found such that the posterior sampling rate was above 30% for all parameters. Note that we have to use a transformation of the parameters such that they are in the correct range, for σ^2 we used an exponential function, for C a logistic transformation and for a a softmax transformation. The change of variables rule was used to update the priors such that the MH worked correctly, this was done by adding the absolute value of the determinant of the Jacobian matrix. Lastly we chose a chain length such that $\hat{R}$ statistic is close to one for each parameter, this indicates that several chains are converging to the same values, so the chains are long enough.

1.1.4 Parametric Logistic (*pL*)

This model is the logistic function adjusted to the data with four parameters found by least square estimation. The model assumes a fixed "S" shape decreasing curve for the responses. This is the usual four parameter logistic fit to the data to minimize a squared error loss function, the model fit is the logistic function given by

$$f(x) = c + \frac{d - c}{1 + \exp b(\log x - \log e)},$$

where c is the asymptotic minimum value, d is the asymptotic maximum value and e is the IC_{50}. Note that we are using $\log x$ in the formula since the fit is done over the logarithm of the doses. The parameters are estimated by minimizing the squared error function $\|y - f(x)\|^2$. There is not an analytical solution to this problem, different numerical minimization algorithms exist for estimating the parameters, our implementation uses the R package `drc`, which internally uses the base R optimizer `optim`. Note that the function is monotonic decreasing unless we have a degenerate fit, in which the adjusted function is a linear fit with non negative slope. Since the function is monotonic and continuous then for any value in the interval $p \in (0, 1)$ the IC_p is the minimum value for which $f(IC_p) = c + p(d - c)$.

1.2 Web Application Options

1.2.1 Input

This section shows how to start using the ENDS to fit parametric and nonparametric drug dose response curves to data. The application accepts as input a *.csv* file with the format as in Table 1 `Workshop` tab and provides different drug-patient characteristics for plotting (Roerink *et al.*, 2018). Once the drug-response data is uploaded or the drug-patient characteristics are selected, click the `Plot` button to compile the graph. Select between viability (decreasing) and inhibition (increasing) responses, the default is viability.

1.2.2 Output

The plots generated can be downloaded in the `Download` tab with the `Download Plot` button. The name can be specified or the default name is the "ENDS". The dots per inch are applicable only to *png* or *jpeg* downloads. The parameters estimated by each of the models and the statistics derived from them can be downloaded in the `Download` tab with `Download Fit` button. The post-processed data can be downloaded in the `Download` tab with `Download Data` button.

name	dose	response 1	…	response m
drug1	d_1	y_{11}	⋯	y_{1m_1}
⋮	⋮	⋮	⋱	⋮
drug1	d_{n_1}	$y_{n_1 1}$	⋯	$y_{n_1 m_1}$
drug2	d_1	y_{11}	⋯	y_{1m_2}
⋮	⋮	⋮	⋱	⋮
drug2	d_{n_2}	$y_{n_2 1}$	⋯	$y_{n_2 m_2}$
⋮	⋮	⋮	⋮	⋮
drugN	d_1	y_{11}	⋯	y_{1m_N}
⋮	⋮	⋮	⋱	⋮
drugN	d_{N_2}	$y_{n_N 1}$	⋯	$y_{n_N m_N}$

Table 1. Data input accepted by web application

1.2.3 Plot Options

The plot provides the following selective options:

Models:

- **Nonparametric Spline (*npS*):** Simple linear spline connecting the mean responses.
- **Nonparametric Monotonic (*npM*):** Is the nonparametric spline with a non-increasing constraint added, by taking recursively the means of the previously accumulated responses until it is non-positive.
- **Nonparametric Bayesian (*npB*):** Specifies a Bayesian hierarchical model which fits a mixture of normal cumulative distribution functions centered at the doses as the basis functions.
- **Parametric Logistic (*pL*):** The usual four parameter logistic model fit by weighted least squares to the data.

Indices for npS:.

- **Point Samples:** If dataset consists of multiple samples, these are shown in the plot.
- **Min-max bands (MMB):** Linear connection of the maximum values for each dose. Same for minimum values. This could be paralleled with the confidence interval in nonparametric setting.
- **Empirical Viability Band (EVB):** It is generated by connecting the sides of the step function generated by the adjacent mean lines, which are the horizontal line that at the mean of each consequent pair of responses. It is used to visualize the variability of the samples within this bands.
- **Drug Span Gradient (DSG):** It is the linear regression fit over the mean responses, the gradient angle θ is given in the plot.
- **Absolute Efficiency Degree (AED):** For dose x_{i+1} is the angle of the slope of the spline from dose x_i to x_{i+1}.
- **Relative Efficiency Degree (ARD):** Is AED$-\theta$, where θ is the angle of the DSG. If the angle is in $(0, 90)$ say the dose x_i is **Negative Relative Dose (NAD)** and otherwise it is a **Positive Relative Dose (PRD)**.
- **Maximum Effective Dose (MED):** Is the dose that compared to its previous dose, exhibit the most descent in terms of the slope (measured in degrees) of the respective section of the spline function of the fit. This is, MED is the next dose for which the minimum AED is observed.
- **Concave Ratio (CR):** Defined as $CR = \#PRD/\#NRD$.

Options:

- **Show Statistics:** Show in corner of plots IC, AUC, MSE and DSG, by default are shown.
- **Median/Mean:** Mean or the median of the responses at that dose, by default it is mean.
- **Outliers Kept:** If not selected then samples outside of 2 standard deviations from mean are removed from data.
- **Viability over 100:** If not selected then values above 100 are replaced with 100.
- **Dose dependent AUC:** If not selected then AUC is calculated with a sequence of integers from 1 to the number of samples as the x-values, else with the doses as the x-values.
- **Spectral choice of IC:** By default set on fifty, this option allows a free choice of the IC value between zero to one hundred.

1.3 Comparative Analysis

For all the data found in (Roerink *et al.*, 2018), we have fitted each of the four models npS, npM, npB and pL to each combination of drug, patient, treatment and sample. For npB we followed the aforementioned procedure of fixing the knots of the function at the doses and choosing the variance parameter of the Normal CDF from a grid of values including the maximum likelihood estimate, as the one which minimized the squared error, and then computed the posterior parameters with the Metropolis-Hastings algorithm for 10,000 iterations. We found the IC_{50}, MSE and AUC for each of the models which are shown in the Figure 1.3. Note that we have included a box plot within the violin plots to showcase where the median, first and fourth quantiles are, and outliers are marked with strong dots. Outliers are those that reside outside the first quantile minus 1.5 times the interquantile range, or the third quantile plus 1.5 the interquantile range.

Using a Shapiro test we reject the hypothesis of normality, so we use the nonparametric Wilcoxon test, to compare whether the mean of models are statistically different from each other. The results of the tests are shown in the Tab. 2, 2, and 4. In summary the IC_{50} of npS and npM are slightly smaller than the pL. The mean squared error of npS is the smallest as the mean is the statistic that minimizes the mean squared error, and so its MSE is different statistically from other models, the npB has a significantly bigger MSE than the pL. The AUC are similar in all the models.

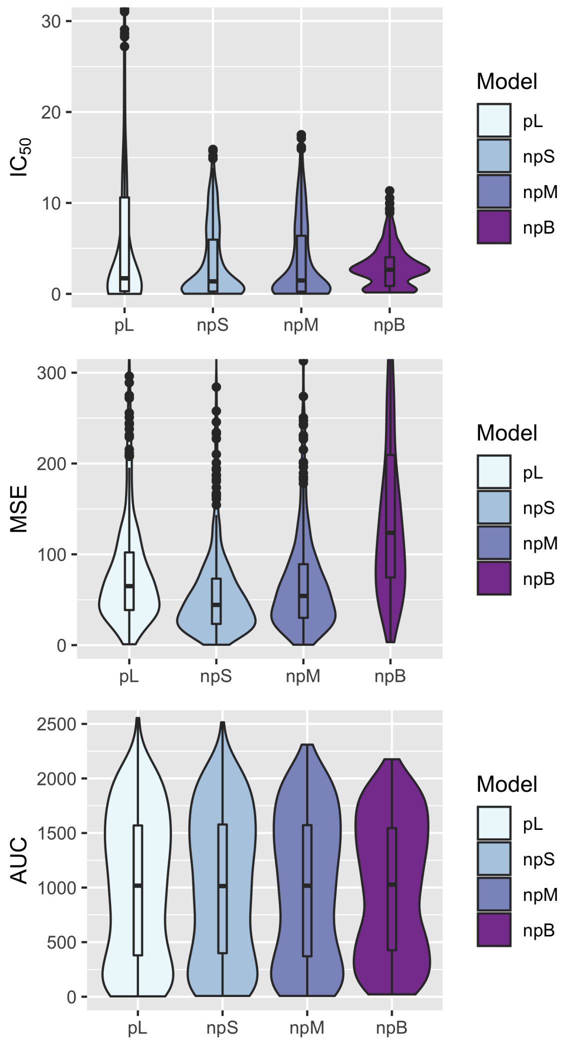

Fig. 1. Violin plots of the IC_{50}, MSE and AUC of the pL, npS, npM and npB models.

IC_{50}	pL	npS	npM	npB
pL	1	0.03*	0.04*	0.42
npS		1	0.77	0**
npM			1	0.01**
npB				1

Table 2. Wilcoxon test p-values for the IC_{50}

MSE	pL	npS	npM	npB
pL	1	0***	0**	0***
npS		1	0**	0***
npM			1	0***
npB				1

Table 3. Wilcoxon test p-values for the MSE

AUC	pL	npS	npM	npB
pL	1	0.87	0.96	0.89
npS		1	0.82	0.99
npM			1	0.87
npB				1

Table 4. Wilcoxon test p-values for the AUC
